# Social determinants of prescribed and non-prescribed medicine use

**DOI:** 10.1186/1475-9276-9-12

**Published:** 2010-05-04

**Authors:** Ferran Daban, M Isabel Pasarín, Maica Rodríguez-Sanz, Anna García-Altés, Joan R Villalbí, Corinne Zara, Carme Borrell

**Affiliations:** 1 CIBER Epidemiología y Salud Pública (CIBERESP), Spain. (C/Doctor Aiguader, 88 1a Planta), Barcelona (08003), Spain; 2 Servei de Salut Comunitària, Agència de Salut Pública de Barcelona, (Pl. Lesseps 1) Barcelona (08023), Spain; 3 Departament de Ciències Experimentals i de la Salut, Universitat Pompeu Fabra, (C/Doctor Aiguader, 88), Barcelona (08003), Spain; 4 Servei de Sistemes d'Informació Sanitària, Agència de Salut Pública de Barcelona, (Pl. Lesseps 1) Barcelona (08023), Spain; 5 Adjunt a Gerència, Agència de Salut Pública de Barcelona, (Pl. Lesseps 1) Barcelona (08023), Spain; 6 Direcció de Farmàcia, Regió Sanitària de Barcelona, (Esteve Terradas 30, Edifici Mestral), Barcelona (08023), Spain

## Abstract

**Background:**

The aim of the present study was to describe the use of prescribed and non prescribed medicines in a non-institutionalised population older than 15 years of an urban area during the year 2000, in terms of age and gender, social class, employment status and type of Primary Health Care.

**Methods:**

Cross-sectional study. Information came from the 2000 Barcelona Health Interview Survey. The indicators used were the prevalence of use of prescribed and non-prescribed medicines in the two weeks prior to the interview. Descriptive analyses, bivariate and multivariate logistic regression analyses were carried out.

**Results:**

More women than men took medicines (75.8% *vs*. 60% respectively). The prevalence of use of prescribed medicines increased with age while the prevalence of non-prescribed use decreased. These age differences are smaller among those with poor perceived health. In terms of social class, a higher percentage of men with good health in the more advantaged classes took non-prescribed medicines compared with disadvantaged classes (38.7% vs 31.8%). In contrast, among the group with poor health, more people from the more advantaged classes took prescribed medicines, compared with disadvantaged classes (51.4% vs 33.3%). A higher proportion of people who were either retired, unemployed or students, with good health, used prescribed medicines.

**Conclusion:**

This study shows that beside health needs, there are social determinants affecting medicine consumption in the city of Barcelona.

## Introduction

Several studies conclude that, apart from health needs, there is a pattern of social variables which define a particular usage of medicines such as age and gender [1-10]. There is somewhat less agreement over the role played by other social variables, such as socio-economic position, access to the health system, employment status or educational level [2,4,6,11-15].

Spain has a National Health Service (NHS) financed mainly by taxes, which provides universal and free health care coverage [16-18]. Even so, for pharmaceutical products there is a system of cost-sharing whereby the user pays 40% of the cost of medicines prescribed by an NHS doctor. However, for certain groups medicines are free: those in hospital, people who are retired older than 65, disabled or have suffered an occupational accident. Patients with chronic diseases pay 10% (until a fixed quantity of 2,95€ maximum) of the cost of medicines, when these are explicitly prescribed by NHS physicians. For medicines which are either not prescribed, or prescribed by a private doctor, the user pays 100% of the cost [17].

According to Spanish Law, a prescription is required for medicines that present a danger either directly or indirectly to health [19]. But sometimes, Spaniards get and take medicines that need a prescription without having the doctor prescription [20,21]. Regarding non-prescribed drugs, a study conducted in Southern Spain found that 13% of medicines requiring prescription are sold in pharmacies without prescriptions [22].

The principal form of access to the NHS is via Primary Health Care centres (PHC) [23]. Beginning in the mid-1980s, the PHC system in Spain was progressively reformed throughout the country (in Barcelona this reform began in 1984, the process being completed in 2003) [24]. As part of the PHC reform, important improvements were made in the quality and effectiveness of health services following the principles of Alma-Ata. As a result of this reform health professionals now work as a team, they use medical records in health centers and are employed full-time; previously, physicians worked alone, some working only 2 hours per day. The reform has been evaluated in different studies and many of these have arrived at very positive conclusions regarding several economic and health aspects [25-28]. Moreover, this reform increased the quality use of prescribed medicines [29-31]. It is important, for the present study, to point out that the public health care system in Barcelona during the period 2000-01 was offering different typologies of PHC, depending on the degree of implementation of the reform process. Moreover, 30% of the population acquires private medical insurance to complement the NHS with elective services or more convenient arrangements for primary care or prenatal care [32,33].

Recently, one study analyzed the patterns of medicine use in the immigrant population resident in Spain [34], but there are no studies analyzing the social patterns of prescribed and non prescribed medicine use among the population of Spain in general. Therefore, the aim of the present study was to describe the use of prescribed and non prescribed medicines in a non-institutionalised population older than 15 years of an urban area during the year 2000, in terms of age and gender, social class, employment status and type of PHC.

## Methods

### Design and source of information

This is a cross-sectional descriptive study. Data were taken from the 2000 Barcelona Health Interview Survey, a cross-sectional survey based on a representative sample of the non-institutionalised population. This survey used stratified sampling by district (Barcelona has 10 districts). The sample of the Barcelona Health Interview Survey consisted of 10,030 persons selected randomly (in each district) from the municipal census. The survey used 4 questionnaires, 3 of which were applicable to people aged over 15 years, namely: questionnaire A (answered by n = 4309 individuals), questionnaire B (n = 4261) and a proxy-responded questionnaire for disabled people (n = 263). The fourth questionnaire dealt with children under 15 years (n = 1197). Data were collected through face-to-face interviews at home between March 2000 and February 2001 by a team of trained interviewers [35]. Persons who did not want to answer the questionnaire (14%) were replaced by another of the same age and sex. The questionnaires included 161 questions related with socio-economic aspects, life-styles, self-perceived health, and health services utilization. The main duration of the interview was 40 minutes.

The questions on medicine use were answered by the interviewee as part of questionnaire B, and formed the basis of the sample used in this study. Women who only took oral contraceptives or medication for menopause (45 in total, 1.1%) were excluded to permit the comparison of results between sexes. The final sample included 4,216 persons.

### Variables

The dependent variable was constructed through the question: "During the last two weeks have you used any of the following medicines? If so, was it prescribed or not?" The list of 13 medicines was: aspirin or similar for pain and fever (analgesics), anxiolytics, medicines to lose weight, antialergics, medicines for cough and flu, antibiotics, vitamins, medicines for stomach conditions, for dizziness, laxatives, menopause hormones (women), anticonceptives (women), other. The dependent variable was the declared use of any of these 13 types of medicines during the two weeks prior to the interview (yes/no). For each medicine used, we recorded whether it had been prescribed or not.

Independent variables were: social class, employment status and type of PHC to which the interviewee's usual general practitioner was associated. Social class was obtained from a Spanish adaptation of the 1980 British Registrar General classification [36]. Class I includes managerial and senior technical staff and free-lance professionals; class II includes intermediate occupations; class III, skilled non-manual workers; class IV, skilled and partly-skilled manual workers; and class V, unskilled manual workers. For analysis purposes, classes were grouped as I-II, III, and IV-V. Subjects in an unpaid job (e.g. women working at home and students) were assigned to the same social class as the head of the household (216 males and 900 females), with the exception of those who had previously worked (including retired and unemployed subjects), who were classified according to their last occupation.

Employment status was categorised as: paid worker, house-person (doing unpaid housework), unemployed, retired or student.

In terms of type of PHC, users were categorised as: 1) users of public health care centres in which the reform had been implemented in the first years (1984 to 1993); 2) users of public health care centres in which the reform had been implemented later on (1994 to 1998); 3) users of public health care centres in which either the reform had not yet been implemented, or was implemented in 1999 or later; 4) users of compulsory or private health insurance schemes; 5) people who had not identified a general practitioner as their usual source of medical care.

We introduced age as a control variable and perceived health status as a stratification variable [37,38]. Age was grouped into four categories for the analysis, separating those older than 65 years, since retired people do not pay for any medicines. Perceived health status was measured through a single question: "Would you say your overall health is very good, good, fair, poor or very poor?" These 5 original categories were re-grouped for the analysis into two levels: "good" which included the first two categories, and "poor" for the remaining three.

Social class was missing or could not be defined in 3.4% of the sample, employment status was missing for 2.4% and type of PHC was missing for 0.6%. Subjects with missing values in these variables were excluded from the descriptive analysis and the multivariable models.

A descriptive analysis was conducted in terms of prevalence of medicine use by age, social class, employment status and type of PHC, stratified by whether prescribed or not, sex and perceived health status. Prevalence by social class were age-standardised, whereas prevalence by employment status and type of PHC were standardised by age and social class, since in Barcelona the PHC reform was implemented first in more deprived neighbourhoods [28]. Standardisation was carried out by the direct method, taking the total sample as the reference population. Subsequently, bivariate logistic regression models were fitted to obtain odds ratio (OR) and associated 95% CI. Lastly, we constructed multivariate logistic regression models in order to determine the factors associated with use of medicines. Due to the absence of any collinearity between the independent variables, all variables were introduced into the model. All models were stratified by whether medicine use was prescribed or not, sex and perceived health status; the 619 (14.5%) persons who took both prescribed and non prescribed drugs at the same time were excluded from these analyses. The Wald test was used to check the statistical significance of the OR. Data were analysed using the SPSS statistical package, version 16.0.

## Results

**Figure 1:**
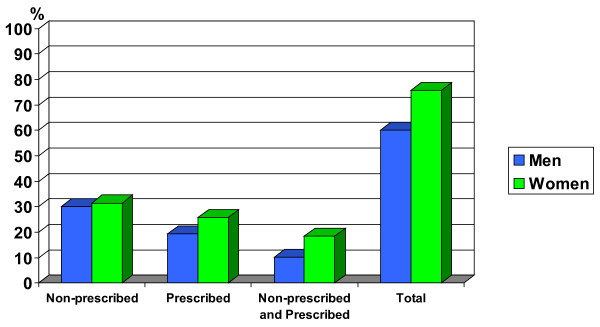
**Percentage of medicine use by type of consumption**. Stratified by men/women. Barcelona Health Interview Survey 2000.

Figure 1 presents the prevalence of declared consumption of any medicines during the two weeks prior to the interview, stratified by sex. 75.8% of women and 60% of men took some medicine, whether prescribed or not.

(show figure 1)

**Figure 2:**
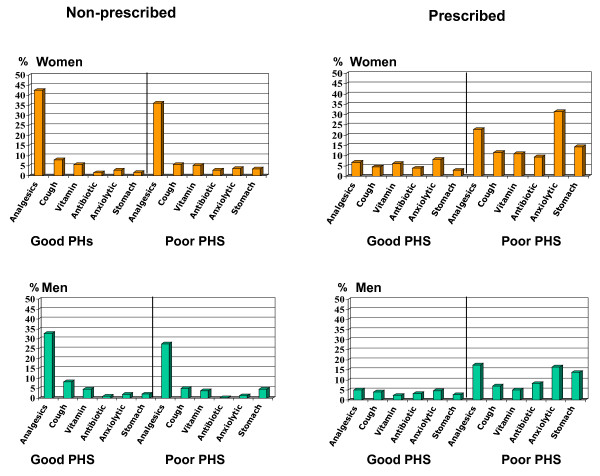
**Percentage of analgesics, medication for cough and flu, vitamins, antibiotics, anxiolytic and medication for the stomach**. Stratified by men/women; good/poor perceived health status and non-prescribed medication/prescribed medication. Barcelona Health Interview Survey 2000. PHS: Perceived health status

Figure 2 shows prevalence of non-prescribed and prescribed use for the most common medicines stratified by sex and perceived health status. In the case of non-prescribed drugs, analgesics and similar drugs are the ones consumed most in both sexes, in both the good and poor health groups. In the case of prescribed medicines, a higher proportion of women with poor perceived health took anxiolytics and analgesics, in comparison with men.

(show figure 2)

Tables 1, 2, 3 and 4 show, for men and women, the distributions of usage of non-prescribed and prescribed medicines in terms of the different study variables. These have been stratified by perceived health status. Results are presented by main independent variables.

### Age

**Figure 3:**
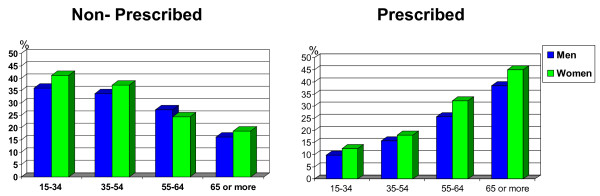
**Percentage of medicine use by age**. Stratified by men/women and non-prescribed medication/prescribed medication. Barcelona Health Interview Survey 2000.

Use of non-prescribed medicines is more common in younger people, whereas use of prescribed medicines is more common among older people (figure 3). However, it should be noted that in the multivariate analysis among those with poor health, these differences are not significant (with the exception of elderly people who use prescribed medicines since they have a higher probability of consuming, OR = 8.63; 95%CI = 1.97-37.77 for men, and OR = 3.13; 95%CI = 1.13-8.63 for women, see tables 3 and 4).

(Show figure 3)

### Social class

The bivariate analysis shows that more men in advantaged classes and with good health took non-prescribed medicines than men in social classes IV-V (38.7% men in social class I-II and 31.8% in social class IV-V). Conversely, the opposite happens among people with poor health: the more disadvantaged classes took more non-prescribed medicines. In the multivariate analysis the same trend can be seen for men with good health (table 1): in the more disadvantaged classes (IV-V) the probability of using non-prescribed medicines is lower (OR = 0.75; 95%CI = 0.57-0.98). Regarding prescribed medicines, in the bivariate analysis there are significant differences only for people with poor perceived health where, in both sexes, more people from advantaged classes (I-II) took prescribed medicines, 51.5% in men and 50.4% in women; in the more disadvantaged classes (IV-V), the corresponding figures were 33.3% in men and 42.2% in women. This trend was maintained in the multivariate model among men (table 3), i.e. that the more disadvantaged classes had lower probability of using prescribed medicines (OR = 0.42; 95%CI = 0.19-0.91). On the other hand, it should be noted that 47.7% of women with poor health in the more advantaged classes were prescribed anxiolytics, whereas in the more disadvantaged classes the figure was 29.1% (results not shown in the tables).

**Table 1:** Number of men who use non-prescribed medicine (n), prevalence of non-prescribed medicine use in % and bivariate and multivariate association between non-prescribed medicine use during the preceding 15 days and independent variables in men, by perceived health status.

	Good perceived health status					Poor perceived health status				
Variables	n	**Prev**.	Bivar OR	Mvar OR	95% CI	n	**Prev**.	Bivar OR	Mvar OR	95% CI
**Age**										
15-34	239	37.0	1	1		7	19.3	1	1	
35-54	192	36.9	0.99	0.94	0.72-1.24	16	17.3	0.87	0.85	0.30-2.39
55-64	51	27.4	*0.64	0.59	0.39-0.89	26	28.3	1.64	1.84	0.65-5.15
> = 65	47	22.6	*0.49	0.41	0.19-0.87	17	9.2	0.42	0.61	0.14-2.61
Total	528	33.9				66	16.4			
**Social class**										
I-II	190	38.7	1	1		6	9.2	1	1	
III	139	30.7	*0.71	0.72	0.54-0.95	14	12.4	1.41	0.88	0.29-2.64
IV-V	195	31.8	*0.73	0.75	0.57-0.98	45	18.6	*2.27	1.36	0.48-3.81
**Employment status**										
Salaried employees	365	33.0	1	1		34	21.4	1	1	
Unemployed men	31	31.0	0.91	1.11	0.69-1.79	7	18.3	0.82	0.62	0.23-1.68
Retired men	55	34.3	1.05	1.21	0.61-2.39	22	12.1	*0.51	0.52	0.17-1.58
Students	73	31.2	0.92	0.89	0.62-1.28	0	0	0	0	0
**Type of PHC**										
PCR 1984-93	96	34.7	1	1		18	17.0	1	1	
PCR 1994-98	93	31.3	*0.85	0.86	0.60-1.22	18	17.1	1.01	1.13	0.50-2.51
No PCR	161	34.7	0.99	1.05	0.76-1.46	22	15.5	0.89	0.77	0.36-1.63
Mutual or private	90	32.6	0.91	1.06	0.72-1.54	4	18.5	1.11	0.54	0.15-1.94
No usual PHC	87	37.1	1.11	1.24	0.84-1.82	4	22.3	*1.41	1.40	0.38-5.15

Barcelona Health Interview Survey, 2000.

PHC indicates primary health care; n, number of cases; 95% CI, 95% confidence interval; Bivar OR, bivariate odds ratio; Mvar OR, multivariate odds ratio; PCR, primary care reform.

* p value < 0.05

**Table 2:** Number of women who use non-prescribed medicine(n), prevalence of non-prescribed medicine use in % and bivariate and multivariate association between non-prescribed medicine use during the preceding 15 days and independent variables in women, by perceived health status.

	Good perceived health status					Poor perceived health status				
Variables	n	**Prev**.	Bivar OR	Mvar OR	95% CI	n	**Prev**.	Bivar OR	Mvar OR	95% CI
**Age**										
15-34	242	41.8	1	1		14	31.7	1	1	
35-54	232	40.6	0.95	1.07	0.81-1.41	28	21.7	0.61	0.61	0.24-1.53
55-64	47	27.0	*0.51	0.58	0.38-0.90	26	20.8	0.57	0.65	0.24-1.72
> = 65	51	20.4	*0.35	0.54	0.31-0.95	60	16.7	*0.43	0.58	0.20-1.64
Total	572	36.3				128	19.5			
**Social class**										
I-II	147	39.4	1	1		7	13.5	1	1	
III	177	33.4	*0.77	0.82	0.61-1.10	22	17.3	*1.34	1.56	0.58-4.16
IV-V	236	38.7	0.97	1.00	0.75-1.34	86	21.4	*1.74	2.14	0.85-5.36
**Employment status**										
Salaried employees	297	34.8	1	1		38	21.2	1	1	
Housewifes	113	38.1	*1.15	0.97	0.70-1.34	53	14.6	*0.63	0.49	0.26-0.93
Unemployed women	35	46.0	*1.59	0.97	0.61-1.53	5	35.3	*2.02	0.95	0.31-2.93
Retired women	29	20.8	*0.49	0.66	0.35-1.25	26	9.6	*0.39	0.45	0.20-1.01
Students	97	40.6	*1.28	1.37	0.96-1.96	4	15.9	0.71	0.72	0.16-3.19
**Type of PHC**										
PCR 1984-93	101	39.7	1	1		28	14.1	1	1	
PCR 1994-98	118	32.9	*0.74	0.79	0.56-1.11	33	17.9	*1.33	1.58	0.84-2.96
No PCR	155	36.9	*0.88	0.88	0.64-1.23	50	20.8	*1.61	1.49	0.84-2.65
Mutual or private	107	35.1	*0.82	0.91	0.62-1.31	11	18.1	*1.35	1.32	0.56-3.07
No usual PHC	86	43.7	*1.17	1.24	0.83-1.85	7	25.4	*2.08	3.00	1.05-8.55

Barcelona Health Interview Survey, 2000.

PHC indicates primary health care; n, number of cases; 95% CI, 95% confidence interval; Bivar OR, bivariate odds ratio; Mvar OR, multivariate odds ratio; PCR, primary care reform.

* p value < 0.05

### Employment status

More significant differences are found among women than among men, in terms of employment status. In the bivariate analysis, among women with good health, non-prescribed medicine use is less frequent among the retired (20.8%) and paid workers (34.8%). In those declaring poor health, use of non-prescribed medicines was more common among unemployed women (35.3%). In the multivariate analysis, only housewives with poor health follow this trend, maintaining a lower probability of using non-prescribed medicines compared to those in paid jobs (OR = 0.49; 95%CI = 0.26-0.93). Use of prescribed medicines, among women in good health (table 4) is more common among students and those retired (27.1% and 26.4% respectively), whereas among those declaring poor health it is more common among retired women (56.1%) and less common among those unemployed (20%). It should be noted that in the case of prescribed medicine use, retired people take more anxiolytics, regardless of whether they reported good health or poor (11.7% and 24.1% respectively). In the case of men in good health, as in women, students and retired people stand out as the groups using prescribed medicines more often (22.9% and 22.1% respectively). A lower proportion of retired people in poor health took non-prescribed medicines.

**Table 3:** Number of men who used prescribed medicine (n), prevalence of prescribed medicine use in % and bivariate and multivariate association between prescribed medicine use during the preceding 15 days and independent variables in men, by perceived health status.

	Good perceived health status					Poor perceived health status				
Variables	n	**Prev**.	Bivar OR	Mvar OR	95% CI	n	**Prev**.	Bivar OR	Mvar OR	95% CI
**Age**										
15-34	60	9.2	1	1		7	19.3	1	1	
35-54	68	13.0	*1.46	1.25	082-1.89	26	27.5	1.62	2.43	0.77-7.63
55-64	35	18.7	*2.25	1.88	1.12-3.18	35	37.6	*2.57	3.13	0.96-10.21
> = 65	61	29.6	*4.12	3.37	1.47-7.69	88	48.5	*4.01	8.63	1.97-37.77
Total	224	14.4				156	38.4			
**Social class**										
I-II	62	15.0	1	1		21	51.5	1	1	
III	76	16.1	1.09	1.15	0.79-1.69	54	43.6	*0.72	0.82	0.37-1.81
IV-V	83	13.4	0.87	1.01	0.69-1.49	78	33.3	*0.47	0.42	0.19-0.91
**Employment status**										
Salaried employees	126	13.9	1	1		32	39.8	1	1	
Unemployed men	12	15.0	1.09	1.33	0.69-2.52	10	31.3	*0.68	1.57	0.64-3.85
Retired men	67	22.1	*1.76	1.08	0.51-2.27	88	34.2	0.78	0.85	0.29-2.46
Students	17	22.9	*1.84	0.65	0.34-1.23	0	0	0	0	0
**Type of PHC**										
PCR 1984-93	25	9.4	1	1		44	43.7	1	1	
PCR 1994-98	48	15.7	*1.81	1.95	1.14-3.32	32	37.9	*0.78	0.58	0.29-1.16
No PCR	78	15.8	*1.81	2.02	1.23-3.33	57	38.8	*0.81	0.72	0.40-1.32
Mutual or private	48	17.9	*2.11	2.26	1.29-3.95	17	45.6	1.08	0.60	0.24-1.47
No usual PHC	23	12.2	*1.33	1.42	0.75-2.66	6	31.1	*0.58	0.45	0.14-1.42

Barcelona Health Interview Survey, 2000.

PHC indicates primary health care; n, number of cases; 95% CI, 95% confidence interval; Bivar OR, bivariate odds ratio; Mvar OR, multivariate odds ratio; PCR, primary care reform.

* p value < 0.05

### Type of Primary Health Care (PHC)

A higher proportion of people with good health in areas where the PHC reform was implemented earlier took non-prescribed medicines (34.7% in men and 39.7% in women). Conversely, for those with poor health, the opposite happens among women (table 2), i.e. in areas where the reform was earlier they took less non-prescribed medicines (14.1%). Regarding use of prescribed medicines, it is noteworthy that among people of both sexes with poor health, it is in the areas reformed earlier that we find more use of prescription drugs (43.7% in men and 49.4% in women). However, for men in good health, the opposite is true (table 3), in that earlier reformed areas present a lower proportion of men being prescribed some medicines (9.4%). This latter trend persists in the multivariate analysis.

**Table 4:** Number of women who take prescribed medicine (n), prevalence of prescribed medicine use in % and Bivariate and Multivariate association between prescribed medicine use during the preceding 15 days and independent variables in women, by perceived health status.

	Good perceived health status					Poor perceived health status				
Variables	n	**Prev**.	Bivar OR	Mvar OR	95% CI	n	**Prev**.	Bivar OR	Mvar OR	95% CI
**Age**										
15-34	64	11.2	1	1		13	29.3	1	1	
35-54	86	14.9	1.4	1.41	0.94-2.11	42	32.2	1.14	1.11	0.43-2.87
55-64	47	26.6	*2.89	2.84	1.69-4.77	50	40.5	1.64	1.87	0.70-4.97
> = 65	91	36.6	*4.62	3.42	1.81-6.46	183	51.1	*2.51	3.13	1.13-8.63
Total	288	18.3				288	43.9			
**Social class**										
I-II	58	17.6	1	1		22	50.4	1	1	
III	86	18.5	1.06	0.94	0.64-1.38	52	44.3	*0.78	0.67	0.32-1.39
IV-V	121	16.8	0.94	0.93	0.63-1.37	188	42.2	*0.71	0.65	0.33-1.27
**Employment status**										
Salaried employees	107	15.2	1	1		42	48.1	1	1	
Housewifes	81	13.2	0.85	0.90	0.59-1.38	133	38.1	*0.66	1.10	0.63-1.91
Unemployed women	19	18.4	*1.26	1.44	0.80-2.56	3	20.0	*0.27	0.52	0.14-1.85
Retired women	57	26.4	*2.06	1.63	0.88-3.04	88	56.1	*1.37	0.99	0.51-1.93
Students	21	27.1	*2.07	0.85	0.48-1.52	4	66.7	*2.15	1.59	0.37-6.87
**Type of PHC**										
PCR 1984-93	50	18.2	1	1		73	49.4	1	1	
PCR 1994-98	53	13.6	*0.71	0.76	0.48-1.20	61	42.4	*0.75	0.74	0.45-1.22
No PCR	101	20.3	1.14	1.22	0.81-1.83	110	42.3	*0.74	0.85	0.54-1.32
Mutual or private	53	16.6	0.89	0.80	0.49-1.30	32	43.7	*0.79	0.69	0.36-1.31
No usual PHC	29	15.5	*0.82	0.81	0.47-1.41	8	26.9	*0.37	0.44	0.15-1.27

Barcelona Health Interview Survey, 2000.

PHC indicates primary health care; n, number of cases; 95% CI, 95% confidence interval; Bivar OR, bivariate odds ratio; Mvar OR, multivariate odds ratio; PCR, primary care reform.

* p value < 0.05

## Discussion

The results of this study show differences in medicine use. More women than men consumed medicines in the 15 days prior to the interview. By age, clear trends are seen in the form of use in both sexes: consumption of prescribed medicines increases with increasing age, while the opposite occurred for non-prescribed medicines. These age differences diminish among people who declared poor health and took prescribed medicines. In terms of social class, a higher percentage of men with good health in the more advantaged classes took non-prescribed medicines compared with disadvantaged classes (38.7% vs 31.8%). In contrast, among the group with poor health, people of more advantaged classes took more prescribed medicines compared with disadvantaged classes (51.4% vs 33.3%). Regarding employment status, a higher proportion of people with good health who were retired, unemployed or students, used prescribed medicines. It should also be noted that anxiolytics use was proportionally higher among retired women regardless of perceived health status.

### Gender and age-related trends

In reference to gender and age, other studies have found similar results [1-9]. Studies conducted in Europe and the USA also showed that medicine use is more frequent among women. Despite this, according to the literature, these differences diminish among older people [5], and are non-existent among children [9]. The higher frequency of use among women has been associated to a poorer perceived health status compared to men [6,8,9]. Another closely related hypothesis involves the existence of greater morbidity, compared to men, leading to higher medicine usage [3,9]. A Swedish study showed that women took more prescribed analgesics than men. Apart from confirming hypotheses mentioned above, they also affirmed that women have less social stigma about admitting to feeling pain, something which influences their analgesics use [8]. Finally, a study conducted in Switzerland claims to have found a tendency involving gender of the health professional in prescribing to women: female doctors prescribed more psychotropics to women than male doctors [10]. Comparing with reports in the literature, in our health interview survey a higher proportion of women presented worse perceived health status and more chronic conditions. It is important to note that, after stratification by perceived health status, we observed that in both strata women took more medicines than men. Regarding the relationship between age and gender, in our study we did not observe a reduction of differences in medicine use between men and women aged over 60 years.

In terms of the influence of age on medicine use, our results coincide with the literature, in that more elderly people used prescribed medicines while more younger people used non-prescribed. Elderly people, due to their high morbidity, go more often to health services and hence have a higher probability of getting a prescription. Conversely, younger people can cover their needs without a medical visit, since most common medicines do not need a prescription: analgesics and similar drugs, medicines for coughs and colds, and vitamins. It should be pointed out that use of analgesics and similar drugs is also high among the elderly but through prescription probably because acetylsalicylic acid is used more for cardiovascular diseases. In contrast, among young people, it is used more as an analgesic. One of the studies conducted in Sweden shows that the effect of age disappears for prescribed medicines after stratification by perceived health status [8]. In our study we also observe that differences in prescribed medicine use diminish with age among those with poor health. We believe that in a health system where access to care is universal, people with poor perceived health go to their doctor regardless of age. Still, it should be noted that, even for poor health, differences in medicine use persist at more advanced ages. The elderly (65 years and over) have the highest morbidity rates however, there is no cost-sharing for them when taking prescribed medicines, something which could influence their higher use.

### Social class and employment status-related trends

Some studies have related medicine use with social class and employment status. Several studies conclude that people not working (pensioners or disabled) [2,11], as well as others with low income [2,12], take more prescription medicines. It is necessary to take into account the healthy worker bias, whereby workers tend to have better health than non workers [39] and consequently that they take less medicines. A less favourable socio-economic situation is intimately related with poor perceived health status and, in countries with a NHS such as Spain, thus leading to greater health services utilization [32] and consequently greater consumption of prescribed medicines [4]. Still, a Danish study, looking at the use of statins by socio-economic position, found that men of high socio-economic positions who presented cardiovascular disease took more prescribed statins than those of low socio-economic position [11]. The authors explain this social inequality as due to a possible influence of the cost-sharing involved of 25%, or to a possibly greater conscientiousness of people in advantaged classes regarding preventive treatments. In regard to social class, our results frequently differ from the literature except for the Danish report. We observed that men with good health in the more advantaged classes (I-II) use more prescribed medicines compared to men of social classes IV-V; and those with poor health in the more disadvantaged classes obtained a lower probability of being given a prescription compared with men of advantaged classes. We believe that our results cannot be explained by cost-sharing, since the class differences in prescribed medicine usage, for good and poor health, persist among elderly people. Among those with good health, 34.4% of people in the more advantaged classes took prescribed medicines, vs. 30.6% of people in disadvantaged classes; for those reporting poor health, the corresponding figures were 58.2% vs. 47.2% respectively (results not shown in the tables). Our results could be explained by a greater proportion of people among the more advantaged classes having double-coverage (around 50% among people of social class I-II compared to less than 20% among people of social class IV-V, depending on sex and health status) and therefore more access to the private specialist [32,33]. Access to the general practitioner can not be an explanation because several studies describe that a higher proportion of people of social classes IV-V visit their general practitioner compared to classes I-II [32].

Regarding employment status, our results largely confirm other reports [2,11]. Among people with good health of both sexes, more retired people, unemployed people and students consumed prescribed medicines compared to paid workers. This phenomenon could be explained because, in the face of lesser need, health service access is less among the working population. Conversely, among those with poor health, the differences are not so clear.

In reference to anxiolytic medicines, this study showed that the prevalence of women with poor health who took anxiolytics was higher than the prevalence of men. Several studies claim that a greater proportion of women aged over 35 years take anxiolytic and antidepressant medicines [1,9,10,40]. They mainly attribute this phenomenon to various factors: a) a higher prevalence of depressive and anxiousness dysfunctions among women [9]; b) a tendency for doctors to prescribe more psychotropic drugs to women [10,40]; c) other social attributes such as women's malaise, principally among women in disadvantaged classes, deriving from the burden of domestic work [40].

### Type of PHC-related trends

Finally, regarding the degree of implementation of the PHC reform in Barcelona, we did not find any significant differences in the multivariate analysis. However, in the bivariate analysis we observe, among those reporting poor health, a greater proportion of men and women receiving prescriptions in areas reformed earlier. This could be due to a higher utilization of these services by the community as a result of various phenomena: a) better care thanks to the reform's better screening [24,26] and b) the reform was first implemented in more deprived areas of Barcelona where the population tended to have worse health [32,33].

## Limitations

This study refers to an urban population, different patterns of medicine use are probably to be expected in rural populations [41]. Another limitation of the study is that in some categories, such as the categories of "mutual or private" or "no usual PHC", we have considered the results not to be relevant due to small numbers. A similar limitation was observed in the "prescribed and non-prescribed" category. The number of people within this category was too small and hence we excluded them from the bivariate and multivariate analysis.

## Conclusions and recommendations

In conclusion, this study has shown that there is a social pattern in medicine use in Barcelona. More women than men consumed medicines, more elderly people took medicines that have been prescribed while more young people took medicines which had not been prescribed. In terms of social class, a higher percentage of men with good health in the more advantaged classes (I-II) took non-prescribed drugs (mainly analgesic and similar drugs) compared with those of disadvantaged classes, whereas in this group with poor health, in the more advantaged classes took prescribed medicines compared with disadvantages classes.

More deep knowledge about the reasons behind this social pattern, as the one obtained in qualitative research [41], should help to reduce such inequalities. Moreover, future studies ought to focus on the appropriateness of those prescriptions.

## Key points

• There is a pattern of social variables which define a particular usage of prescribed and non prescribed drugs: age, sex and perceived health status appear to play an important role.

• In terms of social class, a higher percentage of men with good health in the more advantaged classes took non-prescribed medicines compared with those of disadvantaged classes (38.7% vs 31.8%), whereas in this group with poor health, in the more advantaged classes took prescribed medicines compared with disadvantages classes (51.4% vs 33.3%).

• Considering only people with good health, retired people, unemployed women and students all consumed more prescribed medicines compared to paid workers.

## Competing interests

The paper has not been published elsewhere nor submitted for publication to another journal. We have neither conflicts of interest nor sources of funding apart from our own organizations.

## Authors' contributions

All authors designed the study. FD carried out the analyses and drafted the different versions of the manuscript. MIP and MR-S supervised the data analysis. MIP and CB supervised the different versions of the manuscript. All authors contributed to the different drafts. All authors read and approved the final manuscript.
